# Age is no barrier: predictors of academic success in older learners

**DOI:** 10.1038/s41539-017-0014-5

**Published:** 2017-11-15

**Authors:** Abbie-Rose Imlach, David D. Ward, Kimberley E. Stuart, Mathew J. Summers, Michael J. Valenzuela, Anna E. King, Nichole L. Saunders, Jeffrey Summers, Velandai K. Srikanth, Andrew Robinson, James C. Vickers

**Affiliations:** 1 Wicking Dementia Research & Education Centre, University of Tasmania, Hobart, Australia; 2 Population Health Sciences, Deutsches Zentrum für Neurodegenerative Erkrankungen, Bonn, Germany; 3 Sunshine Coast Mind and Neuroscience - Thompson Institute, University of the Sunshine Coast, Birtinya, Australia; 4 Regenerative Neuroscience Group, Brain and Mind Research Institute, University of Sydney, Camperdown, Australia; 5 University of Tasmania, Hobart, Australia; 6 Research Institute for Sport and Exercise Sciences, Liverpool John Moores University, Liverpool, UK; 7 Peninsula Clinical School, Central Clinical School, Monash University, Melbourne, Australia; 8 Department of Medicine, Peninsula Health, Melbourne, Australia; 9 Menzies Institute for Medical Research, University of Tasmania, Hobart, Australia

## Abstract

Although predictors of academic success have been identified in young adults, such predictors are unlikely to translate directly to an older student population, where such information is scarce. The current study aimed to examine cognitive, psychosocial, lifetime, and genetic predictors of university-level academic performance in older adults (50–79 years old). Participants were mostly female (71%) and had a greater than high school education level (*M* = 14.06 years, *SD* = 2.76), on average. Two multiple linear regression analyses were conducted. The first examined all potential predictors of grade point average (GPA) in the subset of participants who had volunteered samples for genetic analysis (*N* = 181). Significant predictors of GPA were then re-examined in a second multiple linear regression using the full sample (*N* = 329). Our data show that the cognitive domains of episodic memory and language processing, in conjunction with midlife engagement in cognitively stimulating activities, have a role in predicting academic performance as measured by GPA in the first year of study. In contrast, it was determined that age, IQ, gender, working memory, psychosocial factors, and common brain gene polymorphisms linked to brain function, plasticity and degeneration (*APOE*, *BDNF*, *COMT*, *KIBRA, SERT*) did not influence academic performance. These findings demonstrate that ageing does not impede academic achievement, and that discrete cognitive skills as well as lifetime engagement in cognitively stimulating activities can promote academic success in older adults.

## University: who performs better in later life?

Mental activity and select cognitive domains are better predictors of academic performance in later life than previous education or intelligence. Dr. David Ward and a team of researchers from the University of Tasmania and other Australian institutions examined which aspects of older adults predicted performance in a range of university courses. Those who performed better had engaged in more mental activities throughout midlife, such as playing musical instruments or learning a new language, and possessed better memory and language abilities. Previous education only had a limited impact on academic performance, and other factors such as age, intelligence, and gene variations linked to cognition were unrelated to performance. These findings indicate that many of the assumed barriers to education in later life are weaker than anticipated, and highlight opportunities for further education across the lifespan.

## Introduction

There has been recent interest in predictors of educational engagement and attainment.^1^ However, research has typically focused on predictors of academic performance in adolescents and young adults,^2^ and an understanding of the factors associated with academic performance in older adults is lacking. This is despite the proportion of people aged over 60 years growing more rapidly than any other age group, which has prompted an increase in the numbers of older adults undertaking university study.^3^ Not only would an understanding of these factors lead to more effective promotion of academic engagement in later life, but later-life education also has relevance to public health, as such engagement may represent an intervention to reduce population dementia prevalence.^4^

There has been substantial research into which factors predict academic success, with intelligence historically reported as the strongest predictor.^5^ However, more recent findings indicate a modest and variable relationship between intelligence and academic performance (*r* = 0.13–0.60),^6^ suggesting that a substantial proportion of variance in academic success is determined by other factors.^5–7^ Further research has demonstrated an array of other factors that are positively associated with academic performance, such as verbal and emotional intelligence, motivation, and social support.^8–10^ In addition, symptoms of depression have been shown to predict a decrease in academic performance,^11^ and females have been found to outperform males,^12^ but meaningful differences between the sexes are not always detected.^13^

Much of the literature refers to such predictors of academic achievement among young adults; however, these research findings do not always generalise to older adults. For example, grades in high school have been found to be a reliable predictor of academic success for young adults, but not for mature-age university students.^14^ In this regard, the relative importance of predictors of academic performance may change across the lifespan, and factors that may be inapplicable in young adulthood (e.g., occupational attainment) might show relevance in older adults. In addition, there is substantial heritability of cognitive function across the lifespan,^15^ and common genetic polymorphisms that affect cognitive and/or brain function in older age may also account for variance in academic performance. Specifically, genetic polymorphisms of *APOE*,^16^ *BDNF* Val66Met,^17^ *KIBRA*,^18^ *COMT* Val158Met,^19^ and *SERT* 5-HTTLPR^20^ have each been reported to impact upon later-life cognitive performance, risk of cognitive decline, or brain plasticity.

The longitudinal Tasmanian Healthy Brain Project (THBP) was established to determine whether engaging older adults in university education might assist in building resilience to ageing-related cognitive decline and dementia.^21^ Early results indicate that the education intervention does result in measurable increases in proxy-estimated cognitive reserve.^22^ This cohort is uniquely positioned to allow the investigation of specific factors that might mediate academic success for older adults, and whether ageing-related processes affect academic performance. In this study, we aimed to assess the capacity of a range of cognitive, psychosocial, lifetime, and genetic factors to predict university-level academic performance as measured by a 7-point grade point average (GPA). We hypothesised that more years of previous education, higher lifetime engagement in cognitively stimulating activities, higher cognitive ability, and greater social connectedness are associated with higher GPA scores. Further, that older age, higher symptoms of anxiety and depression, and carriage of putative detrimental genetic polymorphisms are associated with lower GPA scores.

## Results

Sample characteristics are displayed in Table 1. On average, participants had a greater than high school education level, had above-average intelligence, and the majority were female. Participants were mostly studying on a part-time basis, were predominantly enrolled in courses within the Faculty of Arts, and on average received university academic results within the credit-distinction range. As determined by a one-way ANOVA, there was no difference in GPA score (*F*_(1,332) = _0.292, *p* = 0.589) between males (*M* = 5.63, *SD* = 1.03) and females (*M* = 5.57, *SD* = 0.94). There was also no difference in GPA score (*F*_(2,331)_ = 0.431, *p* = 0.650) between participants enrolled in the Faculty of Arts (*M* = 5.63, *SD* = 0.89), the Faculty of Science (*M* = 5.71, *SD* = 1.22), or other university faculties (*M* = 5.56, *SD* = 1.06). Those who opted to participate in the genetic substudy were older (*M* difference = 1.91 years, *p = *0.008), had higher Lifetime of Experiences Questionnaire (LEQ) midlife specific scores (*M* difference = 1.09, *p* = 0.036), and had higher language processing performance (*M* difference = 0.28, *p* = 0.011) than those who did not provide samples for genetic analysis. In each case, the magnitude of the difference between the mean values was small (Cohen’s *d* < 0.30).

**Table 1:** Descriptive statistics for all study variables

Variable	*N*	Mean	SD
Demographic			
Age	334	59.64	6.53
Gender (male/female %)	334	29/71	
Prior education (years)	334	14.06	2.76
University study			
Grade point average	334	5.61	1.00
Equivalent full-time study load (%)	334	50.95	33.75
Faculty enrolment (arts/science/other %)	334	47/14/39	
Lifetime experience			
LEQ young adulthood specific	333	16.05	8.22
LEQ young adulthood non-specific	333	24.95	5.52
LEQ midlife specific	332	18.64	4.72
LEQ midlife non-specific	332	24.54	5.68
Cognitive function			
WAIS full-scale IQ	333	119.96	13.24
RAVLT 1–5 total	334	53.84	8.71
LM I immediate recall total	334	48.67	8.22
LM II delayed recall total	334	30.42	6.22
PAL first trial memory score	333	18.51	3.29
Digit span	334	18.68	3.95
Letter-number sequencing	334	11.81	2.38
SWM between errors	332	25.10	18.16
SSP length	332	5.81	1.21
Stroop trial C	332	25.73	7.33
RVP A’	333	0.92	0.05
TMT trial B	333	58.50	20.36
WAIS vocabulary	334	57.24	5.38
WAIS comprehension	334	26.37	3.21
Boston naming test	334	57.79	2.50
Psychosocial function			
LSNS family	334	19.71	5.46
LSNS neighbours	334	10.29	5.55
LSNS friends	334	17.25	5.37
HADS anxiety	334	5.09	3.01
HADS depression	334	2.30	2.16
*Genetic*			
*APOE* (ε4 carrier/non-carrier %)	278	32/68	
*BDNF* Val66Met (Met carrier/non-carrier %)	280	34/66	
*KIBRA* (T carrier/non-carrier %)	279	53/47	
*COMT* Val158Met (Val carrier/non-carrier %)	213	70/30	
*SERT* 5-HTTLPR (*S* carrier/non-carrier %)	260	70/30	

*LEQ* Lifetime of Experiences Questionnaire, *WAIS* Wechsler Adult Intelligence Scale, *RAVLT* Rey Auditory Verbal Learning Test, *LM* Logical Memory test, *PAL* Paired Associates Learning test, *SWM* Spatial Working Memory test, *SSP* Spatial Span test, *RVP* Rapid Visual Processing test, *TMT* Trail Making test, *LSNS* Lubben Social Network Scale, *HADS* Hospital Anxiety and Depression Scale, *APOE* apolipoprotein E, *BDNF* brain-derived neurotrophic factor, *KIBRA* kidney and brain expressed protein, *COMT* catechol-O-methyl transferase, *SERT* serotonin transporter

**Fig. 1:**
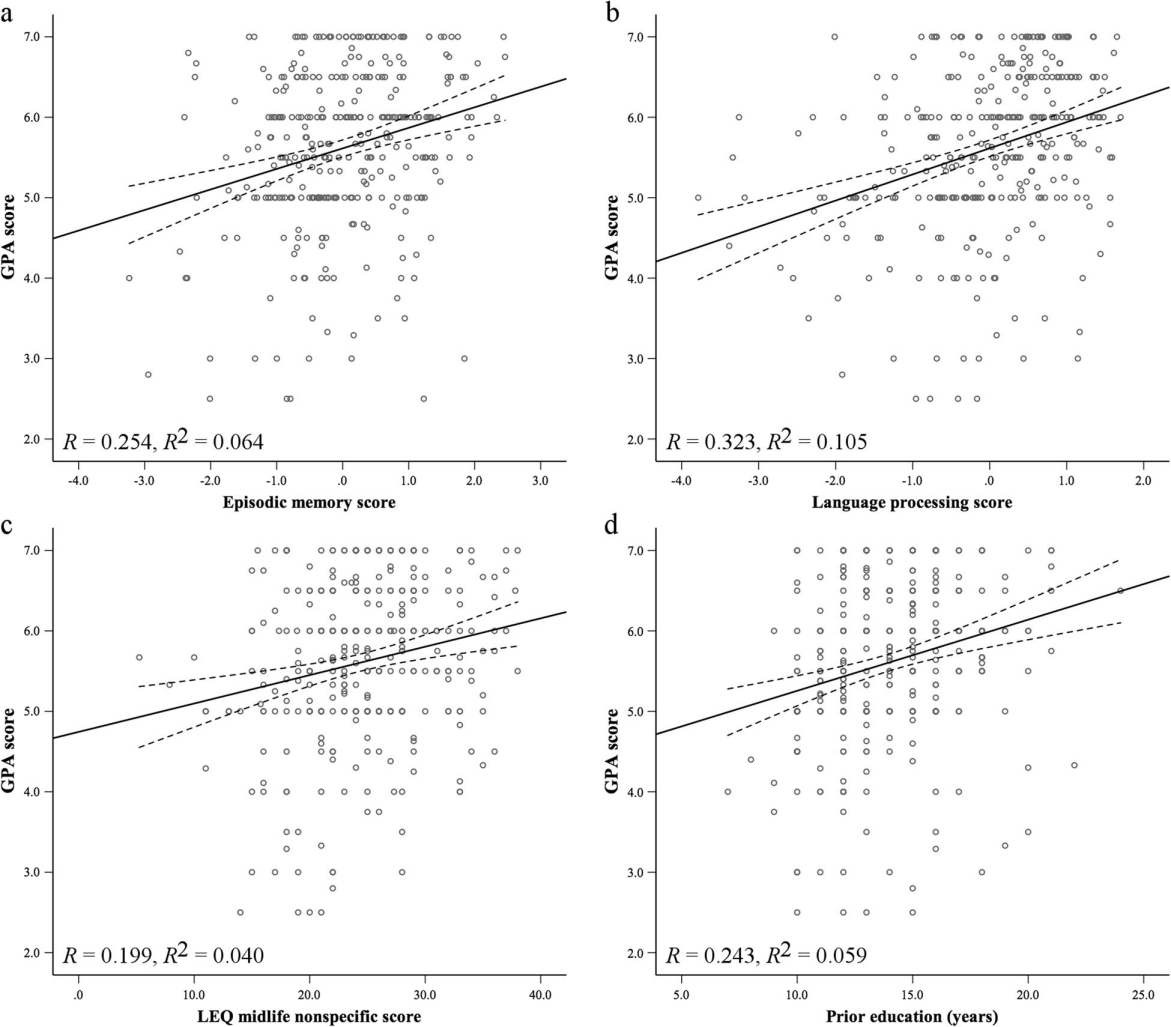
Scatter plots with line of best fit (95% confidence interval) showing significant relationships from the final model and GPA score for **a** episodic memory, **b** language processing, **c** LEQ midlife non-specific, and **d** years of prior education

**Fig. 2:**
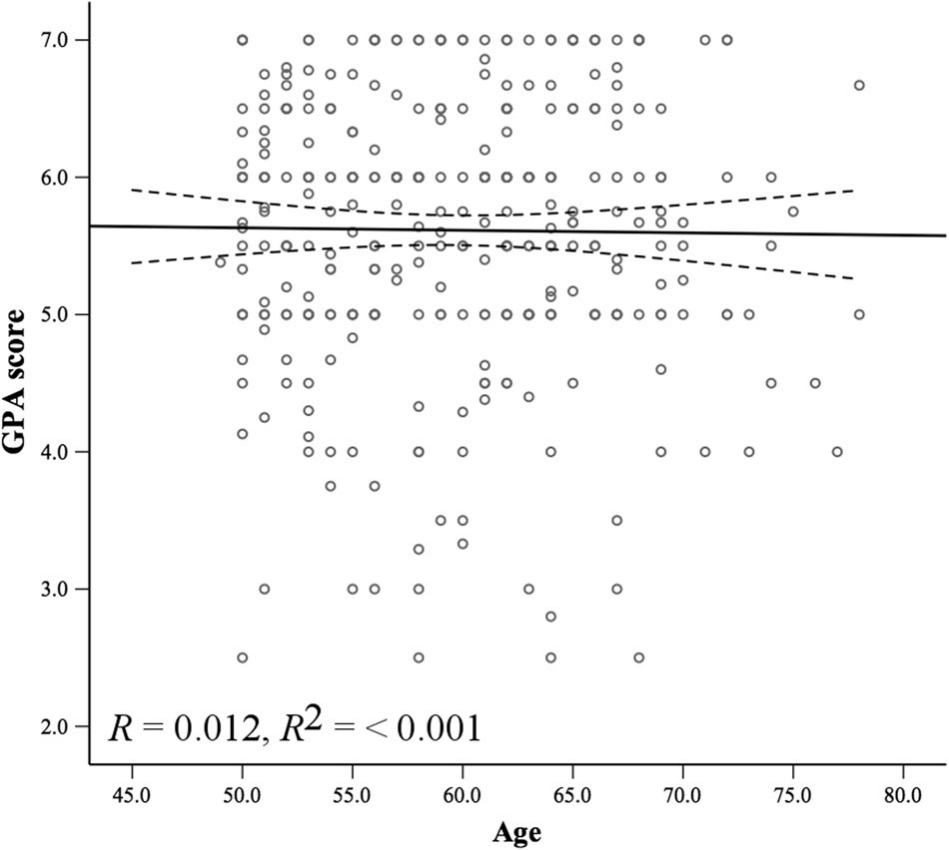
Scatter plot with line of best fit (95% confidence interval) showing non-significant relationship between age and GPA score

An initial multiple linear regression based on all primary predictors of GPA was first conducted in the subgroup of participants who consented to genetic analysis (Table 2). This produced a significant regression equation (*F*_(19,161)_ = 3.254, *p* < 0.001, adjusted *R* ^2^ = 0.192), with four primary predictors significantly associated with GPA (episodic memory, working memory, LEQ midlife non-specific activities). Non-significant predictors of GPA were removed from the model and, as the initial regression identified that no gene polymorphism was significantly associated with GPA, a separate hierarchical multiple linear regression was conducted within the full sample (Table 3). Within step 2 of the model, the secondary predictors of GPA (age, years of prior education, equivalent full-time study load (EFTSL)) were entered. This resulted in an increased model fit when predicting GPA (Δ*F*_(3,321)_ = 3.139, *p* = 0.026, total adjusted *R* ^2^ = 0.186). The predictive capacity of the model increased by 2.3% of the variance in GPA due to the inclusion of secondary predictors, and working memory was no longer significantly predictive. The final significant predictors of GPA, in order of strength of association, were language processing, LEQ midlife non-specific activities, episodic memory, and years of prior education (Fig. 1). Neither age (Fig. 2) nor study load accounted for a significant proportion of the residual variance in GPA.

**Table 2:** Summary of initial multiple regression analysis for all primary predictors of GPA (*N* = 181)

Category	Predictor	*B* (95% C.I.)	S.E.	*β*	*t*	*p*	*sr* ^2^
Lifetime experience	LEQ young adulthood specific	−0.001 (−0.020, 0.018)	0.010	−0.007	−0.090	0.928	0.000
	LEQ young adulthood non-specific	0.024 (−0.007, 0.054)	0.015	0.131	1.533	0.127	0.011
	LEQ midlife specific	0.026 (−0.005, 0.056)	0.016	0.122	1.651	0.101	0.012
	LEQ midlife non-specific	0.042 (0.013, 0.070)	0.015	0.235	2.857	0.005	0.036
Cognitive function	WAIS full-scale IQ	0.001 (−0.013, 0.014)	0.007	0.007	0.080	0.936	0.000
	Episodic memory	0.197 (0.036, 0.358)	0.081	0.194	2.423	0.017	0.026
	Working memory	0.184 (0.020, 0.349)	0.083	0.192	2.217	0.028	0.022
	Executive function	0.083 (−0.091, 0.257)	0.088	0.090	0.943	0.347	0.004
	Language processing	0.210 (0.038, 0.382)	0.087	0.203	2.407	0.017	0.026
Psychosocial function	LSNS family	−0.004 (−0.031, 0.023)	0.014	−0.023	−0.285	0.776	0.000
	LSNS neighbours	0.001 (−0.027, 0.028)	0.014	0.003	0.041	0.967	0.000
	LSNS friends	−0.014 (−0.045, 0.017)	0.016	−0.071	−0.905	0.367	0.004
	HADS anxiety	−0.017 (−0.070, 0.036)	0.027	−0.050	−0.64	0.523	0.002
	HADS depression	0.012 (−0.061, 0.086)	0.037	0.026	0.334	0.739	0.000
Genetic	*APOE*	0.127 (−0.153, 0.406)	0.142	0.063	0.893	0.373	0.004
	*BDNF* Val66Met	−0.019 (−0.301, 0.264)	0.143	−0.009	−0.131	0.896	0.000
	*KIBRA*	0.123 (−0.149, 0.395)	0.138	0.064	0.894	0.373	0.004
	*COMT* Val158Met	0.046 (−0.248, 0.340)	0.149	0.022	0.308	0.759	0.000
	*SERT* 5-HTTLPR	0.206 (−0.081, 0.494)	0.146	0.099	1.416	0.159	0.009

*C.I.* confidence interval, *sr* ^2^ semi-partial correlation squared, *LEQ* Lifetime of Experiences Questionnaire, *WAIS* Wechsler Adult Intelligence Scale, *LSNS* Lubben Social Network Scale, *HADS* Hospital Anxiety and Depression Scale, *APOE* apolipoprotein E, *BDNF* brain-derived neurotrophic factor, *KIBRA* kidney and brain expressed protein, *COMT* catechol-O-methyl transferase, *SERT* serotonin transporter

**Table 3:** Summary of final multiple regression analysis for significant primary predictors from the initial model and secondary predictors of GPA (*N* = 329)

Step	Predictor	*B* (95% C.I.)	S.E.	*β*	*t*	*p*	*sr* ^*2*^
1	LEQ midlife non-specific	0.034 (0.016, 0.051)	0.009	0.191	3.802	< 0.001	0.036
	Episodic memory	0.150 (0.042, 0.258)	0.055	0.148	2.739	0.007	0.019
	Working memory	0.094 (−0.016, 0.203)	0.056	0.092	1.679	0.094	0.007
	Language processing	0.263 (0.154, 0.371)	0.055	0.257	4.772	< 0.001	0.058
2	LEQ midlife non-specific	0.031 (0.014, 0.049)	0.009	0.177	3.515	0.001	0.031
	Episodic memory	0.165 (0.055, 0.276)	0.056	0.164	2.950	0.003	0.022
	Working memory	0.111 (−0.002, 0.223)	0.057	0.109	1.928	0.055	0.009
	Language processing	0.213 (0.100, 0.326)	0.057	0.208	3.709	< 0.001	0.034
	Age	0.010 (−0.007, 0.027)	0.009	0.064	1.151	0.250	0.003
	Years of prior education	0.042 (0.003, 0.081)	0.020	0.114	2.146	0.033	0.011
	Equivalent full-time study load	−0.002 (−0.005, 0.001)	0.002	−0.077	−1.506	0.133	0.006

*C.I.* confidence interval, *sr* ^2^ semi-partial correlation squared, *LEQ* Lifetime of Experiences Questionnaire

## Discussion

This study examined factors associated with university-level academic performance in older adults. In 24 possible predictors, which spanned demographic, lifetime experience, cognitive function, psychosocial, and genetic characteristics, we found that engagement in non-specific cognitive activities in midlife, cognitive performance in both episodic memory and language processing domains, and years of prior education were positively associated with GPA. Strikingly, up to the eighth decade of life, age itself was not associated with academic performance, nor were any of the assessed genetic polymorphisms that had previously been linked to ageing-related cognitive performance, decline, or brain plasticity. These findings were consistent with some of our hypothesised relationships (i.e., positive associations of cognitive ability, engagement in cognitively stimulating activities, and years of previous education with GPA), but many hypothesised relationships were not detected (i.e., GPA negatively associated with age, detrimental genetic polymorphisms, and symptoms of anxiety/depression, but positively associated with greater social connectedness).

Higher-functioning older adults were likely to self-select into the THBP, but this level of functioning is likely representative of most older adults who enrol into university-level education. Despite this, a substantial proportion of the current sample (33%) had not completed greater than 12 years of previous education at study entry, indicating a likely lack of experience with tertiary study. It is possible that this high level of functioning in part accounts for the lack of a negative association of age with GPA, but given that we examined academic performance across a substantial age range (50–79 years old), these findings remain encouraging for older learners. Other predictors that did not demonstrate associations with academic performance were IQ and psychosocial factors (i.e., social networks, symptoms of depression and anxiety). This is in contrast to previous research, where IQ, sex, symptoms of anxiety, and social connectivity were shown to influence academic performance in adolescent to young adult learners.^9,11–13^ In combination, these results indicate that predictors of academic performance are not static across the lifespan, and that the importance of certain factors in earlier life may be attenuated with advancing age. Notably, “mature aged students” have been shown to attain better academic results at university study than their younger peers,^12,13,23^ although the mean age of mature aged students in these studies was 20–30 years, which is in contrast to that of the current study (approximately 60 years old).

In this study, IQ was not a predictor of success in older learners. This finding is consistent with some previous work in this field, which reported that the strength of the relationship between IQ and academic performance declines throughout young adulthood.^24,25^ Although one explanation for this effect relates to students already having been selected for higher levels of education based on their intelligence (i.e., restriction of IQ range in those undertaking complex study^26^), this explanation may not explain the present findings; in our sample of older university students, substantial variance in IQ scores remained (WAIS-III full-scale IQ *M* = 119.96, range = 85–155, interquartile range = 17). In addition, other examinations in primarily young adults have reported a substantial range in correlations (*r* = 0.13–0.60) between intelligence and academic performance.^5–7^ Although IQ was not a significant predictor of GPA in our study, we did find that the specific cognitive domains of language processing and episodic memory were predictive. This indicates that discrete cognitive abilities, rather than broader intelligence, are important to academic performance in older adults. Language processing is relatively preserved with ageing,^27^ and would be central for comprehension tasks and communication as part of university study. Innate ability in episodic memory, which is the ability to encode and remember information and experiences,^28^ would also logically assist in the learning of course-related information.

While we did not find GPA to be associated with specific occupational attainment involving cognitive complexity in midlife, we did find GPA to be associated with midlife engagement in cognitively stimulating leisure activities. In the final model, midlife engagement in cognitive activities was the second strongest predictor of academic performance, and accounted for almost three times of the variance in GPA than did previous years of education. Therefore, a major finding of this investigation is that the level of prior engagement in cognitively stimulating leisure activities is a stronger predictor of later-life success in university education than an individual’s history of engagement in education. This result could suggest that it would be erroneous for a cognitively active older adult to assume that their sparse history of education is a limiting factor when considering university-level education. However, level of engagement with cognitive activities in midlife likely reflects differences in personality traits, with personality factors potentially influencing the degree to which someone pursues such activities. Therefore, personality factors might mediate the positive association of midlife cognitive engagement and GPA. Multiple meta-analyses have highlighted the importance of personality characteristics when examining predictors of academic performance,^7,29^ with greater conscientiousness, and potentially greater openness to experience, associated with better grades. Although the present study examined many possible predictors across a range of categories, data relating to personality factors were unavailable.

The genes selected for investigation included genetic polymorphisms with well-described influences on cognitive function, brain plasticity, and risk of ageing-related cognitive decline (*APOE*,* BDNF* Val66Met, *KIBRA*, *COMT* Val158Met, *SERT* 5-HTTLPR). The observation that none of these gene variations influenced academic performance suggests that genes linked to ageing-related neurodegeneration, as well as neural plasticity, have a low impact on success in purposeful mental activity such as university study. Very large population-based genome-wide association studies have indicated an influence of gene variations on educational attainment in early-life, albeit with very small effect sizes.^30,31^ Therefore, this set of results implies that a candidate gene approach to identifying genetic predictors of academic success in later life may not prove to be successful.

It is important to acknowledge limitations in the current study, in that participants were of a higher than average intellectual capacity than the broader community, were relatively healthy, and did not possess clinically significant symptoms of anxiety or non-treated depression. The predictors identified in the current study collectively explain approximately 19% of variance in GPA; over 80% of variance in GPA is due to factors other than those examined here. Contextual factors (i.e., learning environment) and intrinsic factors, such as student learning approach, motivation, personality, and self-efficacy, have been shown in other investigations to predict GPA for younger adult students,^12,23,32^ but data relating to these variables were not available in this investigation. In addition, a further limitation of the current research was the use of GPA as the sole measure of academic performance. GPA may suffer from grade inflation due to the effect of studying at different levels despite similar academic performance,^33^ which may result in distributions that feature values that cluster within the upper grade range.^34^ However, a fundamental issue in the use of GPA as a measure of academic performance is its reliance on the assumption that grades reflect course intended learning outcomes. Contributions to a student’s GPA from non-constructively aligned units may not necessary reflect the student’s acquisition of core course content, and may be further skewed by other inappropriate assessment and marking practices.^35,36^ Despite this, GPA remains an available and widely used measure of academic performance.^34^

With ageing of the world population, older adults are increasingly engaging in university education, whether for interest or career advancement/redirection.^23^ This study shows that ageing—up to the eighth decade of life—is not an impediment, and that specific cognitive functions (episodic memory and language processing capacity), in combination with attributes associated with lifetime engagement in cognitively stimulating activities, contribute toward academic performance. Coupled with the range of null associations with academic performance (e.g., gender, intellectual capacity, genetics), these results highlight significant opportunities for access and participation of older adults with further education. Furthermore, if such engagement promotes further development of cognitive reserve,^9^ then this may contribute to relative resistance to conditions such as dementia.

## Methods

### Participants

Participants were community dwelling older adults who had consented to annual neuropsychological, psychosocial and health testing as part of the THBP, which is an ongoing interventional cohort study into whether later-life tertiary education protects from ageing-related cognitive decline and dementia. Participants were aged between 50 and 79 years at study entry, and were recruited progressively from 2011 to 2014 through a campaign that involved print, radio, television advertising, and community information presentations. Most participants resided within the state of Tasmania, Australia, and were excluded from entry into the THBP if they presented with conditions that may be independently associated with cognitive impairment (e.g. dementia, multiple sclerosis, epilepsy, brain injury, previous significant head injury, poorly controlled diabetes, chronic obstructive pulmonary disease, heart disease, blindness, deafness, psychiatric disorders). No monetary compensation was provided for participation, but participants were eligible to receive a waiver of individual course charges for a study load equivalent to a 12.5% unit per academic year at the University of Tasmania. The THBP has obtained ethics approval from the Tasmanian Human Research Ethics committee and the current study was conducted in accordance with the ethical guidelines of the National Health and Medical Research Council of Australia.

### Materials

Annual cognitive, neuropsychological and psychosocial assessment of participants in the THBP was undertaken using a comprehensive test battery, as fully detailed in Summers et al.^21^ Age, sex, and years of prior education were collected during participants’ baseline assessments through the use of a self-report medical health status questionnaire. This questionnaire also collected information on handedness, height, weight, marital status, and medical conditions, prescription medication use, and drug and alcohol use. A member of the research team with access to the university database collected data relating to participant EFTSL.

### Academic performance

The outcome measure for this study was GPA, which represents averaged academic performance at university. GPA is derived from the mean score obtained from weighted courses that contribute to an individual’s final degree.^1^ GPA is the most widely used measure of academic performance at university, and has good temporal stability.^1^ A member of the research team collected GPA data from the university database, and participants’ GPA was calculated from their academic performance during their first year of study only. We analysed GPA data from participants’ first year of academic study only, due to the availability of follow-up data and potential confounding issues. Specifically, a complete set of GPA data from second (79% of baseline sample available) and third (49% of baseline sample available) years of study was not yet available. In addition, for those with GPA data from time points subsequent to the initial year, this restriction ensured that the relationships between predictors and GPA were not confounded by prior university study due to THBP participation.

### Lifetime experience predictors of GPA

The Lifetime of Experiences Questionnaire was used as an estimate of mental activity across participant lifespan.^37^ For the purposes of this study, we examined specific subscales relating to two epochs: young adulthood (13–29 years) and midlife (30–64 years). The specific subscales estimated educational attainment in young adulthood and occupational attainment in midlife, while the non-specific subscales estimated frequency of engagement in general cognitively stimulating leisure activities.

### Cognitive function predictors of GPA

Cognitive function was assessed using composite cognitive variables of episodic memory, working memory, executive function, and language processing.^21,38^ Specifically, episodic memory was assessed using the Rey Auditory Verbal Learning Test 1–5 total recall, Logical Memory I immediate recall, Logical Memory II delayed recall, and CANTAB Paired Associates Learning first trial memory score. Working memory was assessed using WAIS Digit Span total recall, WAIS Letter-Number Sequencing total recall, CANTAB Spatial Working Memory between errors, and CANTAB Spatial Span length. Executive function was assessed using Stroop trial C, CANTAB Rapid Visual Processing A', and Trail Making Test B. Language processing was assessed using WAIS Vocabulary, WAIS Comprehension, and Boston Naming Test. Wechsler Adult Intelligence Scale III, short form (WAIS-III-SF1),^39^ was used as a measure of full-scale IQ. Detail relating to the reliability and validity of these tests, in addition to a description of the full test battery, is available in Summers et al.^21^

### Psychosocial predictors of GPA

The Lubben Social Network Scale-18^21^ was used as an estimate of social network size and frequency of social activity, with subscales available for activity related to neighbours, family, and friends. The Hospital Anxiety and Depression Scale^40^ was employed to assess the presence of depression and anxiety symptoms.

### Genetic predictors of GPA

Genetic polymorphisms were determined through DNA extraction of saliva samples. The *SERT* 5-HTTLPR polymorphism was determined following an established standard polymerase chain reaction (PCR) method.^41^ Apolipoprotein E (*APOE*; rs429358, rs7412) and brain-derived neurotropic factor (*BDNF* Val66Met) were determined following established one-step amplified refractory mutation system PCR methods.^42,43^ Catechol-o-methyl transferase (*COMT* Val158Met) and kidney and brain expressed protein (*KIBRA*; rs17070145) were determined following established restriction PCR fragment length polymorphism analysis methods.^44,45^

### Procedure

After obtaining informed consent from each subject, all neuropsychological tests and tools were administered in standard conditions by trained examiners following THBP protocol.^21^

### Data analysis

Prior to the main analyses, composite variables for the four domains of cognitive function were first computed following a method described in detail elsewhere.^38^ Briefly, composite scores for episodic memory, working memory, executive function, and language processing were calculated through factor analyses (principal components extraction method) of domain-consistent raw cognitive test scores, with a single component extracted to represent each domain. This method of producing composite scores has the advantage of statistically accounting for shared variance of related cognitive tests, which is absent when cognitive test *z-*scores are averaged. To include genetic polymorphism data in the regression analysis, variables were dummy coded to values of 0 and 1, where a value of 1 indicated carriage of putative detrimental variants (*APOE* ε4, *BDNF* Met, *COMT* Val, *KIBRA* T, *SERT* short). This method of dummy coding is commonly used to allow for the predictive capacity of bivariate factors to be analysed in regression models.^46^ Due to the low frequency of detrimental allele homozygotes (e.g., *APOE* ε4*/*ε4), we were unable to assess the academic outcomes of inheritance of two copies of such alleles.

Data were screened for outliers and assumptions of multiple linear regression were met. The presence of any group differences in mean GPA score were determined for both sex (male, female) and faculty enrolment (faculty of arts, faculty of science, other faculty) through the use of one-way ANOVA. In accordance with the exploratory nature of the study, multiple linear regression analyses were then fitted to quantify the predictive capacity of primary and secondary predictors on GPA scores. Primary predictors of GPA were lifetime experience, cognitive function, psychosocial, and genetic variables. Secondary predictors of GPA were demographic (age, years of prior education) and university enrolment (EFTSL) variables. First, a multiple linear regression equation was fitted for GPA with all of the primary predictors entered concurrently. Next, non-significant primary predictors were removed from the model and a hierarchical regression approach was employed to examine the residual variance in GPA that secondary predictors accounted for.

The initial regression model was fitted using the genetic subgroup of participants (*N* = 181, observed power = 98.73%), but as no gene polymorphisms significantly predicted GPA, the final regression model was calculated using the full sample (*N* = 329, observed power = 99.99%). The recommended ratio of observations to independent variables in multiple regression analyses varies, including ratios ranging from 5:1 to 10:1.^47^ In our analyses, the initial regression model obtained a ratio of 9.5:1, with the identified significant predictors retested within the full-sample regression model, which obtained a ratio of 47:1. With this considered, we deemed our sample size adequate to produce reliable regression coefficients. An alpha value of 0.05 was used for all statistical tests, and alpha corrections for multiple comparisons were not computed. This decision was made as the difficulty in assessing the wide range of predictors in this population resulted in a relatively small sample size for such an exploratory investigation. However, the identification of stable predictors of GPA was ensured through re-examination of initial predictors in the larger sample, and detailed statistics relating to the reliability of regression coefficients (95% confidence interval) and predictor effect sizes (semi-partial correlation squared) are provided to guide the interpretation of any identified effect. The semi-partial correlation squared (*sr* ^2^) value represents the size of loss of explanatory variance (%) in the overall model should a given predictor be removed.^48^ All analyses were conducted within SPSS Statistics version 21 (IBM, Armonk, NY, USA).

### Data availability

The data that support the findings of this study are available from the corresponding author upon reasonable request.
